# Is There Any Effect of Retrograde Intrarenal Stone Surgery on Renal Functions in Childhood?

**DOI:** 10.7759/cureus.46618

**Published:** 2023-10-07

**Authors:** Kubilay Sarikaya, Cengizhan Ayik, Serkan Akpinar, Fatih Celik, Sema Ayvaz

**Affiliations:** 1 Urology, Ankara Etlik City Hospital, Ankara, TUR; 2 Urology, University of Health Sciences Ankara Atatürk Sanatoryum Training and Research Hospital, Ankara, TUR

**Keywords:** complications, renal functions in children, effects of rirs, renal functions, pediatric renal stone treatment

## Abstract

Purpose

In this study, we aimed to demonstrate whether retrograde intrarenal stone surgery (RIRS) performed due to renal stones during the childhood period has any detrimental impact on renal functions.

Materials and methods

We retrospectively analyzed 121 patients aged 2 to 16 years who underwent RIRS for renal stones between March 2018 and February 2023. Data were available for 41 patients. The estimated glomerular filtration rate (eGFR) was computed using the modification of diet in renal disease (MDRD) formula on the day preceding the surgery and the third month after the surgery. We employed the National Kidney Foundation's chronic kidney disease (CKD) classification to categorize the glomerular filtration rate (GFR) into five groups. Preoperative and postoperative eGFR values of pediatric patients were compared by analyzing changes in CKD groups.

Results

Of the patients included in the study, 21 (51.2%) were male, while 20 (48.8%) were female children. No significant difference was found between the preoperative median eGFR and the postoperative median eGFR values (p=0.958). In the 3rd month after surgery, it was observed that 5 (12.1%) patients showed an improvement in their CKD stage, while deterioration was noted in 1 (2.4%) patient. Although a negative correlation was observed between the median eGFR change and both the operation time and the stone volume in Pearson correlation analysis, it was determined that this change did not create a significant difference (p=0.213 and p=0.295, respectively).

Conclusion

Stone surgery conducted with RIRS appears to yield positive outcomes on kidney function in the pediatric population. Nevertheless, being particularly attentive to patients with prolonged operation times and larger stone volumes is essential.

## Introduction

Although kidney stone disease is a relatively prevalent condition among adults, affecting around 3-5% of the population, it is essential to highlight, from an epidemiological perspective, that children constitute only 2-3% of the entire urolithiasis patient population [1]. Hence, treatment should be tailored to each individual, and comprehensive information about all available treatment options, extracorporeal shock wave lithotripsy (ESWL), retrograde intrarenal stone surgery (RIRS), and percutaneous nephrolithotripsy (PCNL) should be communicated to the patient and their parents [2]. Nevertheless, as indicated by our statistical analysis and clinical observations, parents tend to favor ESWL treatment due to their perception of it being safer and less invasive than the other options [3]. In the case of lack of consent, failure, or contraindications for the ESWL procedure, two endoscopic alternatives can be proposed: transurethral RIRS or percutaneous PCNL. Although considered minimally invasive, various studies have demonstrated that the ESWL procedure can negatively affect renal functions in children [4,5]. PCNL is considered a primary option in the pediatric period, similar to adults, due to its effective stone-free rate, particularly in the surgical treatment of renal stones larger than 2 cm [6]. Studies demonstrate the impact of PCNL on renal functions in children [7]. However, according to our research, there is a lack of literature studies examining the impact of the most preferred procedure in childhood renal stone surgery, RIRS, on renal functions in children. Within the framework of RIRS, the flexible ureteroscope is introduced into the upper urinary tract collecting system utilizing the application of water pressure. During endoscopy, irrigation serves to cool the tip of energy-delivering devices and aids in sustaining a clear visual field by displacing blood, stone fragments, and cellular debris [8]. However, it should be noted that this process can result in prolonged distension of renal calyces. Furthermore, delivering laser energy adjacent to or directly onto renal tissue can potentially damage the renal papillae. Taking into consideration the reasons mentioned and the existing gap in the literature, in this study, we aimed to investigate whether the frequently employed RIRS procedure in childhood renal stone surgery has any detrimental impact on renal functions. Our study is the first to examine the impact of RIRS on renal functions in childhood.

## Materials and methods

Following approval from the institutional review board of Keçiören Training and Research Hospital (dated 08.03.2023, protocol no: 2012-KAEK-15/2665), we conducted a retrospective analysis of a total of 123 patients, ranging in age from 2 to 16 years, who underwent RIRS procedures for intrarenal stones at our clinic between March 2018 and February 2023. Data were available for 41 patients. We documented the patient's age, gender, number of stones, cumulative stone diameter, stone localization, and hydronephrosis grade. Preoperative and postoperative evaluations of patients were conducted using computed tomography. Patients with stones located in various parts of the kidney underwent a thorough examination, including complete blood count, urine analysis, urine culture, as well as assessments of urea, creatinine, and routine biochemical parameters prior to the surgical procedure. All procedures were performed in a sterile urine setting and validated by a urine culture conducted five days before the RIRS. When urinary infections or bacteriuria were confirmed, they were treated with the administration of suitable antibiotics for a period of at least five days. Following antibiotic treatment, the urine cultures of these patients were retested, and negative cultures were obtained. Documented data encompassed the mean operative time, fluoroscopy duration, ureteral access sheath utilization, postoperative placement of ureteral stents or double-J (DJ ) stents, hospitalization day, and the count of procedures conducted. The study did not include patients with second-session RIRS, those with a solitary kidney, a history of chronic renal failure, co-existing ureteral stones, ureteral strictures, urinary tract anomalies, or individuals with missing follow-up data. Patients from the prior session who could not establish renal access due to insufficient ureteral calibration and only received DJ catheter placement, subsequently undergoing RIRS 3-4 weeks later, were also included in the study. Perioperative complications were categorized using the modified Clavien grading system [9]. We considered a patient to be stone-free when no residual fragments or <2 mm residual fragments were visible on a non-contrast-enhanced tomography carried out three months after the retrograde flexible ureteroscopy session.

Surgical procedure

Informed consent was secured from all patients prior to the surgeries, and to provide surgical prophylaxis, IV first-generation cephalosporin was administered 60 minutes before the commencement of the procedures. The operations were conducted by six distinct surgeons, all affiliated with the same institution, and the procedures were performed under general anesthesia in a typical flexible ureteroscopy setup. The procedure for patients with previously placed DJ stents was continued after the simultaneous removal of their stents. A 0.035-inch polytetrafluoroethylene-coated wire was carefully inserted into the upper urinary tract under direct visual and using a rigid cystoscope, with a safety wire consistently employed as a standard practice. The ureteral access sheath (Elite Flex®, Ankara, Turkey) was placed in the ureter if possible; in cases where the ureteral access sheath could not be placed, direct entry was made using a flexible ureteroscope via a 0.035-inch guide wire. An RIRS procedure was performed using a 7.5 F flexible ureteroscope (Flex-X2®, Karl Storz, Tuttlingen, Germany). Fluoroscopy control was performed in suspected cases where the ureteral access sheath had slight difficulty as it advanced through the ureter. Laser lithotripsy was carried out with a 270 mm laser fiber (Boston Scientific, AMS/USA) with energy settings ranging from 0.5 to 1.5 Joules and a 5 to 10 Hertz frequency. Following the completion of the surgery, a ureteral stent was implanted in all patients, and the operation duration was defined as the time elapsed from the initiation of cystoscopy to the conclusion of ureteral stent placement. Intraoperative details were carefully recorded, and patients who encountered no complications were released on the initial day after the operation. Patients' control X-ray images were examined in the postoperative follow-up between the 15th and 30th days. After ensuring that there were no residual stone fragments requiring additional intervention, the DJ stents were removed. All laboratory results and screening findings were documented in the follow-up form, and on the third-month follow-up, patients were monitored through CT scans and routine laboratory tests. The minimum follow-up duration for all patients was three months after the surgery.

Renal function evaluation

The assessment of the glomerular filtration rate (eGFR) was determined using the equation developed by the modification of diet in renal disease (MDRD) study group [10]. The patients' preoperative and postoperative 3rd-month eGFR values were determined and recorded. We utilized the National Kidney Foundation's chronic kidney disease (CKD) classification, which divides estimated GFR into the following ranges: ≥90 mL per minute per 1.73 m² (Stage 1), 60-89 mL per minute per 1.73 m² (Stage 2), 45-59 mL per minute per 1.73 m² (Stage 3a), 30-44 mL per minute per 1.73 m² (Stage 3b), 15-29 mL per minute per 1.73 m² (Stage 4), and <15 mL per minute per 1.73 m² (Stage 5) [11]. Any change leading to a more favorable or less favorable GFR group after the surgery was deemed significant.

Statistical analysis

Data were analyzed using SPSS 25.0 (IBM Corp., Armonk, NY, USA) software. The median and minimum-maximum range were used for descriptive statistics. The Mann-Whitney U-test and Kruskal-Wallis test were used for univariate analysis to determine the parameters related to eGFR change. The Pearson correlation test was used to assess the correlation between eGFR change, stone volume, and operation time. A p-value of <0.05 was considered statistically significant.

## Results

The Male/Female ratio was 21/20. Among the children included in the study, 12 (29.3%) were between the ages of 2-6, 12 (29.3%) were between 7-12, and 17 (41.5%) were between 13-16 years old. It was determined that among the stones treated with RIRS, 26 (63.4%) were located in the renal pelvis, 9 (21.9%) in the lower calyx, 3 (7.3%) in the middle calyx, 2 (4.8%) in the upper calyx, and 1 (2.4%) had a multicalyceal placement. The mean stone volume was 210±59 mm^3^. Among the patients, 27 (65.85%) had previously placed DJ stents, while 14 (34.15%) achieved primary renal access without the need for prior DJ catheterization. The preoperative patient data and descriptive findings are presented in Table 1.

**Table 1:** Preoperative patient characteristics and descriptive outcomes. DJ stent: Double-J stent.

Category	Subcategory	N	%
Age (years)	2-6	12	29.3
	7-12	12	29.3
	13-16	17	41.5
Gender	Male	21	51.2
	Female	20	48.8
Stone Location	Renal pelvis	26	63.4
	Lower Calyx	9	21.9
	Middle Calyx	3	7.3
	Upper Calyx	2	4.8
	Multicalyceal	1	2.4
No. of Stones	1	34	82.9
	2	6	14.6
	3	1	2.4
Stone Volume, mean±SD, mm3		210±59	
Prior DJ stent placement		27	65.85
Hydronephrosis	No	10	24.3
	Grade 1	11	26.8
	Grade 2	18	43.9
	Grade 3	1	2.4
	Grade 4	1	2.4

Median operation time was 40 (25-65) minutes, and median hospital stay was 1 (1-6) days. It was observed that a stone-free status was achieved in 38 (92.7 %) out of 41 patients at the end of the operation. According to the Clavien grading classification, while no complications developed in 30 (73.1%) patients, Grade 1 complications developed in 7 (17.0%) patients, Grade 2 complications developed in 3 (7.3%) patients, and Grade 3 complications developed in 1 (2.4%) patient. In addition, no serious complications at the Grade 4 or Grade 5 level were observed in any patient (Table 2).

**Table 2:** Perioperative and postoperative patient outcomes and complications. D-J stent: Double-J stent.

Parameter	Status	Median (minimum-maximum)	N%
Operation time, minutes		40 (25-65)	
Fluoroscopy time, minutes		0 (0-20)	
Hospital stay, days		1 (1-6)	
D-J stent time, days		21 (15-30)	
Ureteral access sheath placement	Yes		35 (85.3)
	No		6 (14.7)
Stone free	Yes		38 (92.7)
	No		3 (7.3)
Complication	No		30 (73.1)
	Grade 1		7 (17.0)
	Grade 2		3 (7.3)
	Grade 3		1 (2.4)
	Grade 4		0
	Grade 5		0

At the postoperative 3rd month, improvement in the CKD scale was observed in 5 patients (12.1%), while deterioration in the CKD scale was noted in 1 patient (2.4%). The preoperative median eGFR value of the patients was 110 (55-123), and at the postoperative 3rd month, the median value of this parameter was observed to be 110 (51-122), with no significant difference detected (p=0.958) (Table 3).

**Table 3:** Preoperative and postoperative CKD groups and eGFR changes in patients. CKD: Chronic kidney disease; eGFR: Estimated glomerular filtration rate.

	Preoperative	Postoperative 3rd month	P-value
CKD groups (n, %)			0.018
Stage 1	35 (85.3)	39 (95.1)	
Stage 2	6 (14.6)	2 (4.9)	
Stage 3	0	0	
Stage 4	0	0	
Stage 5	0	0	
CKD groups (n, %)	-	5 (12.1)	-
CKD stage deterioration (n,%)	-	1 (2.4)	-
eGFR change			0.958
eGFR, median (min-max)	110 (55-123)	110 (51-122)	-1 (-7 to +9)

It was observed that there was a negative correlation between eGFR changes in the preoperative and postoperative 3rd month and stone volume and operation time, but it was determined that this correlation level was not at a level to make a statistically significant difference (p=0.213 and p=0.295, respectively) (Table 4). In the long-term follow-up at 12 months, it was determined that there were no serious complications such as urethral or ureteral stenosis in any patient.

**Table 4:** Analyzing the correlation of eGFR change with operation time and stone volume. eGFR: Estimated glomerular filtration rate.

	Operation Time	Stone Volume
eGFR change		
Pearson	-198	-168
P-value	0.213	0.295

## Discussion

As children with nephrolithiasis represent a patient group at an elevated risk of stone recurrence throughout their lives, it is crucial to promptly assess and manage their condition. Epidemiological studies show that the incidence of urolithiasis in the pediatric age group ranges from 2% to 10% [1,6,12]. It has been observed through studies that the incidence of kidney stones tends to be higher among boys in their initial 10 years and among girls during the subsequent decade of life [13]. The pathophysiological basis of nephrolithiasis can be conceptually categorized into three key aspects. The formation of stones relies on (1) solute concentrations surpassing their solubility in urine, (2) crystallization driven by an imbalance between promoters and inhibitors, and (3) the binding and enlargement of crystals into nephroliths as a consequence of epithelial abnormalities [14]. Common solutes contributing to stone formation include calcium, oxalate, phosphate, citrate, uric acid, and cysteine. Additionally, common inhibitors of stone formation comprise citrate, magnesium, macromolecules, and pyrophosphate [15]. ESWL is often considered the initial treatment choice for pediatric renal stone management, particularly for stones larger than 20 mm. Short-term studies report a stone-free rate of 67-93%, while long-term studies indicate a range of 57-92% [16]. Despite its utility, ESWL has certain limitations, particularly when dealing with resistant stones like cystine or calcium oxalate monohydrate and stones situated in the lower pole. Furthermore, concerns persist about the potential development of diabetes mellitus or hypertension during long-term follow-up after ESWL [17]. Additionally, studies have shown that ESWL may cause certain complications and loss of renal function in children as well as in adults. Yucel S et al., in their study involving 128 pediatric patients, showed the rate of complications requiring rehospitalization in the first week after ESWL [18]. Their study reported an overall success rate of 93.5%. During the first postoperative week, a total of 22 patients (17.8%) experienced complications, with only 19 individuals (15.4%, comprising 12 boys and seven girls) requiring rehospitalization at that time. In another similar study, Villányi KK et al. examined the short-term effects of ESWL on renal functions in a total of 16 children [19]. They have noted a substantial rise in the excretion levels of aspartate transaminase, alkaline phosphatase, lactate dehydrogenase, and beta 2-microglobulin, signifying proximal tubular dysfunction and cellular damage following the ESWL procedure. According to the observations in this study, enzyme levels returned to their baseline within 15 days. Based on their study, they recommend a minimum 15-day gap between two shock wave treatments to ensure functional regeneration. However, they also emphasized the necessity of conducting extensive research on the long-term consequences. One of the most essential treatment alternatives for renal stones in childhood is PCNL [20]. Cicekbilek I et al. investigated the effect of PCNL performed in childhood on renal functions in their study involving 40 patients and 41 renal units [7]. According to this study, out of the 41 renal units examined, new focal cortical defects were observed on (99m)Tc-DMSA scans in four (9.7 %) patients following PCNL. Furthermore, this study demonstrated that the mean creatinine level before PCNL was 1.18 ± 0.45 (ranging from 0.8 to 1.6) mg/dL, which was comparable to 1.16 (ranging from 0.7 to 1.5) mg/dL after the follow-up period (p > 0.05). They concluded that the PCNL in children is both safe and practical for achieving maximal stone clearance. While the stone-free rate following PCNL in children has been documented to reach as high as 68-100%, it is important to note that complications, including fever, sepsis, renal pelvic perforation etc., can still occur post-procedure [21]. Nevertheless, it is important to emphasize that employing adult-size instruments for PCNL in preschool-age children tends to result in a higher complication rate. Hence, adopting specialized equipment, such as small-diameter nephroscopes, is deemed essential for PCNL procedures in this patient age range.

With the routine utilization of a flexible ureterorenoscopy, RIRS has emerged as a significant therapeutic option for renal stones, serving as an effective approach not only in adults but also among pediatric patients. Unsal A and Resorlu B reported the results of a total of 16 (M/F: 9/7) patients who underwent RIRS due to renal stones in childhood [22]. According to their study, it was reported that the median operation time was 52 (30-85) minutes, and the median hospitalization time was 2.1 (1-4) days. In contrast, the total stone-free rate in the same study was reported as 88%. In addition, it was reported in their study that ureteral perforation developed in only one (6%) patient and was healed by insertion of a ureteral stent. In another similar study by Erkurt B et al., the results of a total of 65 children who underwent RIRS in the preschool pediatric age were reported [16]. According to this study, the mean operation time was reported as 46.47+18.27 minutes, and the mean hospital stay was reported as 1.49+1.42 days, while in the same study, the total stone-free rate was reported to be 92.3% at the end of two sessions of RIRS. Furthermore, in the same study, they reported complications in 18 (27.7%) patients as postoperative hematuria (Clavien I) in six (9.2%) patients, postoperative urinary tract infection with fever (Clavien II) in 10 (15.4%) patients, and ureteral wall injury (Clavien III) in two (3%) patients. In our study, median operation time and hospitalization time were found to be compatible with literature data. In addition, our stone-free rate was found to be 92.7%, comparable with the literature data. In our study, it was observed that minimal ureteral damage occurred in only one (2.4%) patient and was treated by placing a ureteral stent without requiring additional surgical intervention. These findings support the idea that the RIRS procedure can be applied effectively and safely in treating renal stones in childhood. Although studies report a high success rate, there is not enough data in the literature on studies showing the effect of RIRS performed during childhood on renal functions. The long-term pressure of the irrigation fluid on the calyceal system and the direct energy transmission of laser energy used in stone fragmentation to the calyceal system may result in a certain level of renal function loss after RIRS [8]. Acute elevation in fluid pressure can pose a serious threat to tubular function. The hydrostatic pressure-induced stretching of renal tubular cells triggers an inflammatory response within the tubular interstitium, marked by the proliferation of macrophages and the accumulation of myoblasts. Changes in tubular cells, in association with the infiltration of macrophages and myoblasts, result in the synthesis of cytokines and growth factors. These biochemical mediators are accountable for provoking apoptosis in renal tubular cells. Consequently, this leads to the emergence of chronic obstructive nephropathy, with tubular atrophy and the loss of nephrons, which are then substituted by interstitial fibrosis [23]. In our study, we revealed that the change in eGFR between the preoperative period and the third postoperative month was not significant. Additionally, in our study, it was observed that there was a significant improvement in the CKD stages of the patients after RIRS compared to the preoperative period. Although it was not statistically significant, our study showed that there was a negative correlation between median eGFR change, median operation time, and median stone size. The lack of statistically significant negative correlation among these factors is likely attributable to the small sample size of our study. This result indicates that the eGFR change will be higher with the prolongation of the operation time in children with large stone size, and the possible loss of renal function may develop more. Consequently, advanced randomized clinical trials are essential to determine the suitable stone dimensions and the most effective operation duration for RIRS. We believe that conducting further studies involving a larger cohort of pediatric patients will provide more definitive and elucidating results on this matter.

Limitations

The most important limitation of our study is its retrospective nature. Given the retrospective nature of our study, it is essential to note that no control group was included. Consequently, we could not perform a comparative analysis by establishing a control group. These outcomes are based on a study with a relatively small sample size, and it is important to underscore that our multivariate analysis did not confirm multiple procedures. On the other hand, the lack of homogeneity in the age groups of the children included in the study and the absence of a measurement method, such as (99m)Tc-DMSA, to separately assess functional changes in both kidneys represent significant limitations of our study. Additionally, the fact that the operations were performed by more than one surgeon in our study may be a confounding factor.

## Conclusions

In conclusion, our study underscores the effectiveness of RIRS as a highly successful and low-risk alternative for treating renal stones in childhood, with a success rate exceeding 90% and a minimal complication rate. Importantly, our findings reveal that in the third month following RIRS, there were no significant alterations in renal functions among pediatric patients. However, it is crucial to note that as stone size increases and the duration of the procedure extends, a more pronounced reduction in estimated glomerular filtration rate (eGFR) levels following RIRS becomes apparent. Consequently, when performing RIRS in the pediatric population, striving for an optimal operation duration is imperative. Additionally, patients undergoing longer procedures should be subject to close postoperative monitoring to promptly detect and address any potential declines in renal function. These insights emphasize the importance of careful consideration and monitoring during RIRS procedures in childhood, ultimately contributing to the safe and successful management of renal stones in this patient group.

## References

[1] Malek RS, Kelalis PP (1975). Pediatric nephrolithiasis. J Urol.

[2] Samotyjek J, Jurkiewicz B, Krupa A (2018). Surgical treatment methods of urolithiasis in the pediatric population. Dev Period Med.

[3] Brinkmann OA, Griehl A, Kuwertz-Bröking E, Bulla M, Hertle L (2001). Extracorporeal shock wave lithotripsy in children. Efficacy, complications and long-term follow-up. Eur Urol.

[4] Reis LO, Zani EL, Ikari O, Gugliotta A (2010). Extracorporeal lithotripsy in children - The efficacy and long-term evaluation of renal parenchyma damage by DMSA-99mTc scintigraphy. Actas Urol Esp.

[5] Lottmann HB, Archambaud F, Hellal B, Pageyral BM, Cendron M (1998). 99mTechnetium-dimercapto-succinic acid renal scan in the evaluation of potential long-term renal parenchymal damage associated with extracorporeal shock wave lithotripsy in children. J Urol.

[6] Kaygısız O, Türegün FA, Satar N (2018). Renal stone composition does not affect the outcome of percutaneous nephrolithotomy in children. World J Urol.

[7] Cicekbilek I, Resorlu B, Oguz U, Kara C, Unsal A (2015). Effect of percutaneous nephrolithotomy on renal functions in children: assessment by quantitative SPECT of (99m)Tc-DMSA uptake by the kidneys. Ren Fail.

[8] Hoarau N, Martin F, Lebdai S, Chautard D, Culty T, Azzouzi AR, Bigot P (2015). Impact of retrograde flexible ureteroscopy and intracorporeal lithotripsy on kidney functional outcomes. Int Braz J Urol.

[9] Lee WJ, Smith AD, Cubelli V, Badlani GH, Lewin B, Vernace F, Cantos E (1987). Complications of percutaneous nephrolithotomy. AJR Am J Roentgenol.

[10] Levey AS, Bosch JP, Lewis JB, Greene T, Rogers N, Roth D (1999). A more accurate method to estimate glomerular filtration rate from serum creatinine: a new prediction equation. Modification of Diet in Renal Disease Study Group. Ann Intern Med.

[11] Acosta-Ochoa I, Bustamante-Munguira J, Mendiluce-Herrero A, Bustamante-Bustamante J, Coca-Rojo A (2019). Impact on outcomes across KDIGO-2012 AKI Criteria according to baseline renal function. J Clin Med.

[12] Van Batavia JP, Tasian GE (2016). Clinical effectiveness in the diagnosis and acute management of pediatric nephrolithiasis. Int J Surg.

[13] Novak TE, Lakshmanan Y, Trock BJ, Gearhart JP, Matlaga BR (2009). Sex prevalence of pediatric kidney stone disease in the United States: an epidemiologic investigation. Urology.

[14] Miah T, Kamat D (2017). Pediatric nephrolithiasis: a review. Pediatr Ann.

[15] Ratkalkar VN, Kleinman JG (2011). Mechanisms of stone formation. Clin Rev Bone Miner Metab.

[16] Erkurt B, Caskurlu T, Atis G (2014). Treatment of renal stones with flexible ureteroscopy in preschool age children. Urolithiasis.

[17] Krambeck AE, Gettman MT, Rohlinger AL, Lohse CM, Patterson DE, Segura JW (2006). Diabetes mellitus and hypertension associated with shock wave lithotripsy of renal and proximal ureteral stones at 19 years of followup. J Urol.

[18] Yucel S, Akin Y, Danisman A, Guntekin E (2012). Complications and associated factors of pediatric extracorporeal shock wave lithotripsy. J Urol.

[19] Villányi KK, Székely JG, Farkas LM, Jávor E, Pusztai C (2001). Short-term changes in renal function after extracorporeal shock wave lithotripsy in children. J Urol.

[20] Schlomer BJ (2020). Urologic treatment of nephrolithiasis. Curr Opin Pediatr.

[21] Desai M (2005). Endoscopic management of stones in children. Curr Opin Urol.

[22] Unsal A, Resorlu B (2011). Retrograde intrarenal surgery in infants and preschool-age children. J Pediatr Surg.

[23] Hamdi A, Hajage D, Van Glabeke E (2012). Severe post-renal acute kidney injury, post-obstructive diuresis and renal recovery. BJU Int.

